# Associations between Wildfire Smoke Exposure and Health-Related Quality of Life: Findings from the Lovelace Smokers Cohort

**DOI:** 10.21203/rs.3.rs-7069099/v1

**Published:** 2025-10-23

**Authors:** Qizhen Wu, Yanhong Huang, Lisa L. Sinclair, Huining Kang, Tyler Eshelman, Maria A. Picchi, Marissa Childs, José M. Cerrato, Yiliang Zhu, Su Zhang, Steven A. Belinsky, Matthew J. Campen, Xi Gong, Shuguang Leng

**Affiliations:** University of New Mexico; University of New Mexico; University of New Mexico; University of New Mexico; University of New Mexico; Lovelace Biomedical Research Institute; University of Washington; University of New Mexico; University of New Mexico School of Medicine; University of New Mexico; University of New Mexico; University of New Mexico; University of New Mexico; University of New Mexico

**Keywords:** Wildfire smoke, PM2.5, black carbon, SGRQ, SF-36

## Abstract

**Background:**

The impact of wildfire smoke (WFS) on air quality across the contiguous US has become geographically widespread. However, the effects of episodic WFS exposure on psychometric measures of mental and physical health remain largely unknown.

**Objectives:**

To assess the associations between WFS PM_2.5_ and black carbon (BC) exposure and psychometric health measures.

**Methods:**

The St. George’s Respiratory Questionnaire (SGRQ) and the 36-Item Short Form Survey (SF-36) were administered to participants in the Lovelace Smokers Cohort in New Mexico to assess psychometric health measures in the past 4 weeks. WFS estimates were calculated against Albuquerque metropolitan area or individual residential addresses for 7-, 15-, 30-, and 60-d prior to questionnaire filling. The associations between exposure and health measures were assessed using linear models.

**Results:**

Significant associations were observed for all psychometric measures with WFS PM_2.5_ and BC exposures estimated for 7-d prior to questionnaire filling. Significant associations remained for WFS exposure estimated up to 30-d prior to questionnaire filling for all SGRQ subdomains and physical health measures of SF-36, but became non-significant for the mental health measures of SF-36 beyond one week prior. Additionally, WFS PM_2.5_ exhibited stronger potency than total ambient PM_2.5_. Male participants, individuals with less than a college education, and those exposed to woodsmoke demonstrated heightened vulnerability to WFS.

**Conclusions:**

Episodic exposure to WFS was associated with worse SGRQ and SF-36 scores, with notable differences in temporal patterns between mental and physical health measures. Our findings also underscore the importance of source-specific risk assessment for air pollution.

## Introduction

Rising temperatures, prolonged droughts, and increasing vegetation desiccation accompanying climate variability are dramatically intensifying the frequency, duration, and severity of wildfires across North America and globally (1–3). Wildfire smoke (WFS) has had a spatially and temporally profound impact on air quality across the contiguous U.S., stalling or reversing multi-decadal declines in fine particulate matter (PM_2.5_) concentrations in 35 states, and contributing significantly to the rise in extremely polluted days (daily PM_2.5_ >35 μg/m^3^) in 18 states since 2012 (4, 5). The impact of WFS on air quality is no longer transient or negligible, as it has contributed an average of 1 μg/m^3^ annually to ambient PM_2.5_ concentrations in the eight most affected states in the western and midwestern U.S. since 2016. Moreover, in high-fire years (2017, 2018, and 2020), WFS contributes up to 5 μg/m^3^ to annual PM_2.5_ concentrations, which is equivalent to roughly half of the total annual average PM_2.5_ from all sources across much of the contiguous U.S. (5). Since 2010, WFS has also significantly increased the black carbon-to-PM_2.5_ ratio in Western U.S., thus could potentially elevate the toxicity of PM_2.5_ (6–9).

Individual wildfire episodes can last anywhere from weeks to months, releasing smoke plumes into the atmosphere that contain large amounts of particles and toxicants. These wildfires, along with the smoke they produce, can significantly affect local air quality and public health. In addition, prevailing winds can carry WFS hundreds or even thousands of miles away from the source. Once the smoke descends, it impacts near-ground air quality, affecting the health of populations far from the original fire sites (2). Therefore, geographic locations affected by WFS can either be near the burning sites or downwind regions, with air quality deteriorating in an episodic manner. This means that when wildfires are active, air pollutants in these regions can increase temporarily, ranging from weeks to months. Acute health effects from extreme WFS exposure can occur within hours or days, including cardiovascular events, respiratory symptoms and exacerbation, eye irritation, and reduced cognitive performance (10–14). Evidence for chronic health effects after multiple rounds of episodic WFS exposure begins to emerge and supports the etiological link of WFS with lung and brain cancer and incident dementia (15, 16). However, evidence supporting the health impacts of episodic WFS exposure, which usually last from weeks to 2–3 months in the contiguous U.S., is very limited. Only one study has reported obstructive airway changes following a 45-day WFS exposure in Seeley Lake, Montana, where daily PM_2.5_ levels averaged 220.9 μg/m^3^. Notably, these airway changes persisted even two years after the exposure (17). Delineating these early changes will inform both acute and chronic health risks, identify vulnerable subgroups, and reveal underlying mechanisms for potential mitigation options.

This study aimed to investigate the associations between episodic exposure to WFS and health-related quality of life (HRQoL), which captures multi-dimensional assessment of physical health, mental well-being, and social functioning. Additionally, we assessed individual traits that may sensitize people for WFS health effects. These analyses were conducted using data from the Lovelace Smokers Cohort (LSC), located in Albuquerque, an arid region frequently impacted by WFS from wildfires originated in Gila, Apache, and Apache-Sitgreaves National Forests due to prevailing southwest winds in warm seasons.

## Methods

### Study population

**Figure 1:**
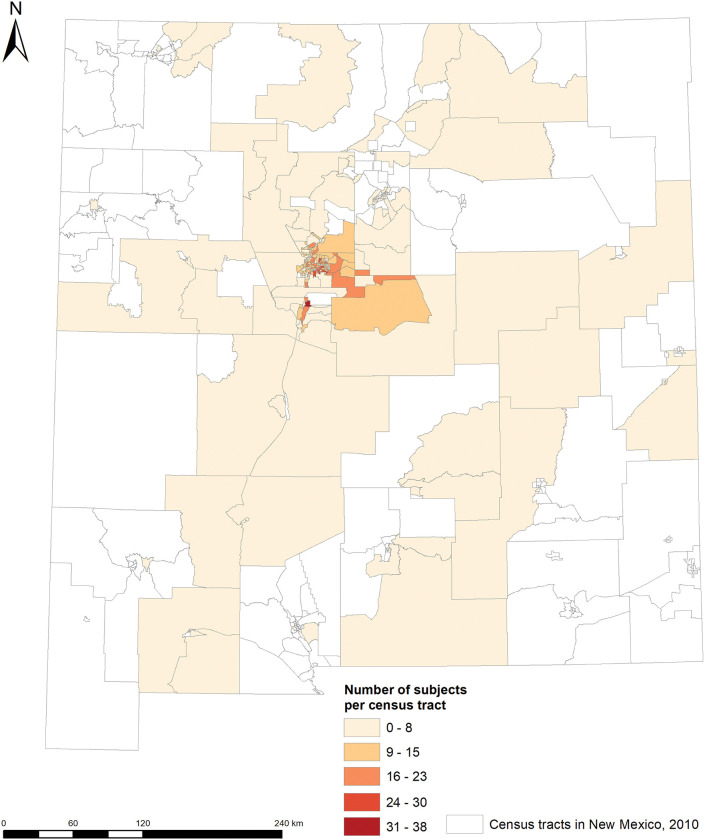
Geospatial distribution of the LSC participants by census track in New Mexico.

The LSC is a longitudinal, population-based volunteer cohort with majority of participants enrolled from the greater Albuquerque area of New Mexico from 2001 to 2017 (Fig. 1). The primary objective of the LSC was to identify biomarkers in sputum and blood for lung cancer and chronic obstructive pulmonary disease (COPD) development. The design of the LSC has been described elsewhere (18–20). Briefly, a total of 2511 participants aged 40 to 75 years, with at least 10 pack-years of smoking and no prior history of lung cancer, were recruited through local newspaper, radio, and television advertisements. At baseline, participants completed a standardized questionnaire on demographics, tobacco smoking, medical history, diet, as well as quality of life measures (the St. George’s Respiratory Questionnaire [SGRQ] and the 36-item short-form health survey [SF-36]). They also underwent pre- and post-bronchodilator spirometry and provided biological samples (blood and sputum). Follow-up visits were conducted approximately every 18 months till November 2017. All participants provided informed consent, and the study was approved by the Western Institutional Review Board.

### Health-Related Quality of Life Measures

Health-related quality-of-life (HRQoL) was assessed using the general health SF-36 questionnaire and the lung disease-specific SGRQ with the recall period of past four weeks (21, 22). The SGRQ total score and its activity, symptom, and impact domain subscores range from 0 to 100, with higher score indicating a worse HRQoL (23). A minimal clinically important difference in SGRQ total score and domain subscores is 4 (24). The SF-36 encompasses eight domains including physical functioning, role physical, role emotional, social functioning, mental health, vitality, general health perceptions, and bodily pain. The SF-36 scores range from 0 to 100, with higher scores indicating better HRQoL (21). Confirmatory factor analysis using R package *lavaan* was used to identify latent constructs representing physical and mental health based on factor structures established in U.S. general populations (25). These two factor scores were then used in subsequent analyses to reduce dimensionality.

### WFS assessment

The majority of the LSC participants were from the greater Albuquerque area including Bernalillo, Sandoval, Valencia, and Torrance counties, thus we used this area (a total of 18,719 km^2^) to estimate WFS exposure. First, we calculated the number of smoke days in periods of 7-, 15-, 30-, 60- days prior to questionnaire filling. The 60-day maximal length was selected because majority of WFS episodes that led to elevation in air pollution in Albuquerque lasted two months or shorter. Smoke days were defined as when the study area was overlapped with satellite-detected smoke plumes by at least 1 km^2^, based on data from the National Oceanic and Atmospheric Administration (NOAA) Hazard Mapping System (HMS). Second, we also calculated cumulative area of the greater Albuquerque area overlapped with WFS plumes for individual time periods to estimate the geographic massiveness of the WFS. Third, we assessed WFS exposure using quantitative metrics such as smoke PM_2.5_ and smoke black carbon (BC) in ambient air. These quantitative metrics effectively captured near-ground pollutant levels, providing a more precise assessment compared to smoke plume-based methods. We took advantage of recent advances in air quality modeling that provided ambient PM_2.5_ and BC data at higher geospatial (1 × 1 km) and temporal (daily) resolution across the contiguous US from 2000 to 2020 (6). We followed Dr. Child’s published methods to estimate smoke PM_2.5_ and smoke BC (4, 5). In brief, smoke PM_2.5_ and BC were quantified as deviation in PM_2.5_ or BC values in smoke days from the median values from non-smoke days in the same month over a three-year period, spanning the year before and the year after. Zero was assigned to smoke days when the subtraction gave negative values, i.e., smoke plumes did not compromise near ground air quality. Average smoke PM_2.5_ and smoke BC in 7-, 15-, 30-, 60- days prior to questionnaire filling were calculated. Additionally, Dr. Childs and colleagues developed a machine learning algorithm to predict smoke PM_2.5_ levels through integrating a comprehensive dataset spanning ground monitoring records, expanded smoke plume data, fire emission inventories, land use and elevation, meteorology, and satellite aerosol measurements (4, 5). This smoke PM_2.5_ dataset covered the contiguous US from 2006 to 2020 and has an excellent prediction accuracy, verified specificity for WFS, and sufficient geo-spatial (10 × 10 km) and temporal (daily) resolution for health linkage study (4, 5). We applied the county-level daily WFS dataset developed by Dr. Childs and colleagues as an alternative exposure assessment in sensitivity analyses.

### Geocoding

We used residential locations to calculate WFS exposure and as sensitivity analyses to validate results from the greater Albuquerque area. Addresses were geocoded with ArcGIS 10.8, standardizing data and matching it to the Albuquerque Street map to assign latitude and longitude. These geocoded locations helped estimate WFS exposure by assigning average smoke PM_2.5_ and BC concentrations based on the corresponding 1 × 1 km grid for each specified time period.

### Statistical analysis

Statistical analyses were conducted in 747 LSC subjects who were enrolled in 2006 (first year when smoke plume data became available) and after and had measures of SGQR and SF-36 at baseline. Linear model was used to assess the associations between WFS measures and SGRQ and SF-36 scores, with adjustment for baseline age, sex, BMI, ethnicity, education level, status and packyears of tobacco smoking, airway obstruction for SGRQ, and baseline comorbidity for SF-36 (19, 26, 27). Due to moderate to high correlations among WFS measures (Supplemental Fig. 1) and among outcomes, instead of setting arbitrary cutoffs for claiming study-wide significance, we chose to weight evidence based on nominal P values as well as consistency across measures and time periods. Interaction analyses were conducted to explore whether the observed associations varied across several candidate factors such as sex, education, current smoking status, woodsmoke exposure (self-reported in response to a question “Have you ever been exposed to woodsmoke for 12 months or longer” as part of the general health survey at study entry of the LSC), chronic mucous hypersecretion, and ethnicity. Interaction analyses were conducted in 7-day WFS – SGRQ or WFS – SF36 associations due to the greatest significance. P values less than 0.1 for the interaction terms were deemed meaningful interactions. These interaction analyses should be viewed as secondary analyses to reduce the issue of multiple comparisons. As a sensitivity analysis, we excluded participants who were followed up during winter (n = 66), as significant WFS events were rare in the Albuquerque area during this season. All analyses were conducted using R (version 4.4.1, Vienna, Austria) in the RStudio (version 2024.9.0.375).

## Results

### WFS and Air Quality

**Figure 2:**
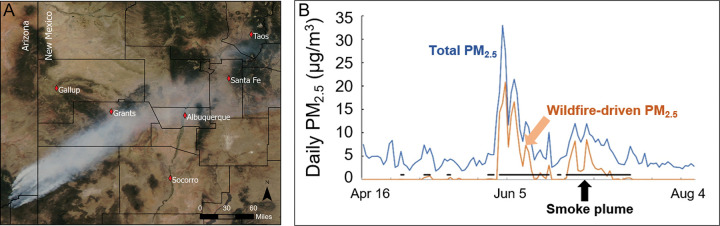
Smoke day, wildfire smoke PM_2.5_, and total ambient PM_2.5_ in Albuquerque in the summer of 2011. A. MODIS satellite image demonstrates actively burning wildfires in Apache-Sitgreaves National Forests and atmospheric transfer of the smoke from the burn sites to central New Mexico. This imagery represents a “true color” band combination of data collected by the MODIS instrument on the NASA Aqua satellite acquired June 6, 2011. Band1=Visible Red (0.620 – 0.670 μm), Band3=Visible Blue (0.459 – 0.479 μm), Band4=Visible Green (0.545 – 0.565 μm). The image represents the three spectral bands scaled to a resolution of 250m per pixel at the equator. B. Wildfire smoke as primary contributor to episodic elevation of ambient PM_2.5_ in Albuquerque in the summer of 2011.

The greater Albuquerque, the catchment area of the LSC was frequently affected by WFS during summer that predominantly originated from the fires in Apache and Gila National Forest around the Arizona and New Mexico border (Fig. 2A) (28). The distance between these fires and Albuquerque metropolitan area is 150 to 200 miles, suggesting aged WFS. The estimated ambient PM_2.5_ and BC concentrations were significantly higher in 474 smoke days versus 3909 non-smoke days (6.55 μg/m^3^ versus 4.14 μg/m^3^ for PM_2.5_ and 0.25 μg/m^3^ versus 0.16 μg/m^3^ for BC). For example, in the summer of 2011, WFS released from the Wallow Fire is the major contributor to elevated ambient PM_2.5_ (Fig. 2B).

### Study Participants and their WFS Exposure

The 747 participants had an average mean (SD) age of 56.9 (9.1) years and included 383 females, 185 Hispanics, and 447 current smokers (Table 1). A total of 479 subjects had received some college education and above. A total of 241 subjects self-reported to be “ever woodsmoke exposure for over a year”. Comparisons between people with and without smoke days in a week prior to questionnaire filling did not identify any significant differences for variables under consideration (Table 1). Moreover, about 400, 540, and 613 subjects were exposed to smoke days in 15-, 30-, and 60- days prior to questionnaire filling (Table 2).

### Associations between WFS and the HRQoL

Significant associations with all four SGRQ scores were identified for WFS PM_2.5_ estimated for 7-, 15-, and 30-day time frames with 1 μg/m^3^ increase associated with > 4 points of increase in activity, symptom, and total SGRQ scores, a clinically significant alteration (Table 3). When using same unit of change (0.05 μg/m^3^) to quantify impacts of WFS on SGRQ scores, BC exhibited over 20-fold stronger potency in affecting SGRQ scores compared to PM_2.5_ (Table 3). Similar temporal patterns were also observed for physical health score of SF-36 except that WFS BC’s impact remains significant for the 60-day time window prior to questionnaire filling (Table 4). However, the impact of WFS on mental health measure was predominantly seen for WFS estimates a week prior to questionnaire filling (Table 4). It was also interesting to note that WFS PM_2.5_ was much more potent (> 2.7 fold) in affecting symptom SGRQ scores and physical health measures compared to total ambient PM_2.5_. Association analyses based on the number of WFS day and area affected by WFS can reproduce majority of the associations seen using WFS PM_2.5_ or BC (Tables 3 and 4).

### Sensitivity analyses

Excluding visits occurring in winter when there were few WFS episodes affecting the greater Albuquerque area did not change the associations observed (Table S1 and S2). Association analyses using WFS measures based on residential addresses reproduced the results seen using WFS measures based on four counties. Moreover, stronger magnitudes of association were observed for symptom score of SGRQ for 7-d and 15-d WFS measures and for mental and physical health measures of SF-36 for 15-d WFS measures (Table S3 and S4). Using four-county based WFS PM_2.5_ measures from Dr. Childs’ study reproduced the temporal patterns of associations with SF-36 measures, however its associations with SGRQ scores were only observed in 7-d WFS measures (Table S5 and S6).

### Effect modification

We conducted interaction analyses to determine whether the WFS – HRQoL associations were modified by several candidate factors (Table S7 - S13). These analyses were conducted using 7-day WFS measures to ensure optimal power as 7-day measures had most significant associations with HRQoL measures. Most consistent effect modifications were identified for “ever woodsmoke exposure for over a year” as subjects with woodsmoke exposure have elevated vulnerability to WFS induced HRQoL changes compared to people who do not have woodsmoke exposure (Table S7). We also found evidence that males and subjects with less than college education were more vulnerable to WFS induced HRQoL changes (Table S8 and S9).

## Discussion

Through linking daily WFS estimates to psychometric measures collected at baseline visits of the LSC members, we identified that episodic WFS exposures significantly deteriorated psychometric measures of multiple health domains. The impact of WFS exposure on SGRQ scores had similar temporal patterns across all subdomains, *i.e.*, respiratory symptoms, limitation in physical activity due to breathlessness, and psychosocial disturbances, with significant associations observed for WFS exposure estimated for 7-, 15-, and 30-d prior to questionnaire filling. However, temporal patterns for the impact of WFS exposure on SF-36 differed by subdomains with impacts seen up to 30-d exposure for physical health measures, while only 7-d exposure for mental health measures. The link between WFS exposures and physical health measures of SF-36 and respiratory quality of life measures by the SGRQ is more likely to be driven by inhalation exposure of WFS and lung deposition of BC particles. Phagocytosis of BC by airway macrophages is a major clearance mechanism in the acinar airway, which triggers persistent cytokine/chemokine secretion and generation of other mediators for downstream pulmonary and extra-pulmonary toxicity (29). Our recent analyses in 88 LSC subjects identified that exposure to elevated PM_2.5_ in the air for over a month due to WFS (summer) or residential wood burning (winter) significantly increased macrophage carbon load (28).

Despite the prevalence and persistence of psychological disorders in communities facing wildfire threats, there remains a notable gap in research regarding the effects of WFS on general mental health in populations not directly affected by the fires, but exposed to smoke that travels long distance in atmosphere. We identified the first human evidence supporting alterations of mental health measures after acute exposure to aged WFS. The time frame for triggering alteration in mental health measures was rather short, *i.e.*, a week prior to questionnaire filling and this impact became non-significant beyond one-week time window of WFS exposure regardless of whether WFS continued or not. A meta-analysis published in 2019 showed that short-term exposure to PM_10_ in the air was associated with the risk of completed suicide at a 0–2d cumulative time lag in meta-analysis (30). There was also some evidence suggesting an association between short-term PM_2.5_ or PM_10_ exposure and depression-related emergency department visits (30). A randomized, double-blind, crossover trial in China demonstrated that use of air purification for 9 days significantly reduced indoor PM_2.5_ and stress hormones (cortisol, cortisone, epinephrine, and norepinephrine) in blood circulation (31). Our animal models discovered brain effects such as neuroinflammation, neurometabolomic alterations, and depression-like behavioral changes after inhalation exposure to WFS for 4 hours/day, every other day, for 2 weeks (7 exposures total) at an environmentally relevant level (448 μg/m^3^ PM_2.5_) (32, 33). Several key mechanisms linking inhalation exposure of nanoscale particles and brain effects have been proposed and include direct enter of PM_2.5_ in brain tissue via the olfactory nerve, circulating mediators and compromised blood brain barrier integrity, and transfer of PM_2.5_ through blood gas barrier and circulation (29, 34). However, it remains unclear whether the associations with mental health measures we observed are due to mechanisms described above due to the rapid attenuation of associations beyond one-week time window of WFS exposure as well as much lower levels of WFS PM_2.5_ in our study population compared to animal exposure study. Further research using blood omics approach in populations with real-world WFS exposure may help decode the mechanisms underlying how episodic WFS exposure affect mental health measures in humans.

Combustion-emitted PM is demonstrated to be more harmful to health than PM from non-combustion sources and the literature shows that estimation of combustion emitted PM using source apportionment or BC outperforms total PM_2.5_ mass levels for evaluating health risks (7, 35). Our analyses provide evidence supporting this premise. The SGRQ scores were most significantly associated with WFS PM_2.5_ rather than with total ambient PM_2.5_. For physical health measures, WFS PM_2.5_ showed 2.7- to 3.4-fold stronger associations compared to total ambient PM_2.5_, although both PM_2.5_ metrics were significantly associated with the outcomes. Stronger potency in inducing lung effects by WFS PM_2.5_ compared to PM_2.5_ from other sources was reported in animal models with lung lavage cytology and lung histology as the outcome (36) and in California populations with respiratory hospitalizations as the outcome (37). Additionally, days with high pollution levels (PM_2.5_ ≥35 μg/m^3^) resulting from WFS were strongly associated with an increased risk of tuberculosis diagnoses in California, unlike those caused by other sources of air pollution (38). All together, these studies suggest that WFS PM may be more toxic than equal doses from other sources. WFS PM_2.5_ is mostly carbonaceous (with 5–20% elemental carbon and at least 50% organic carbon) and has more oxidative potential than ambient urban particulate due to the presence of more polar organic compounds (39, 40). It is therefore imperative to differentiate between smoke and non-smoke PM_2.5_ when assessing impacts on public health. Moreover, WFS BC also demonstrated stronger associations with all health measures compared to WFS PM_2.5_, suggesting BC constituents may be more potent in inducing health effects compared to non-BC constituents in WFS PM_2.5_.

It is also important to note that male participants, those with lower than college education, and those reporting ever woodsmoke exposure were more vulnerable to the adverse physical health impacts of WFS. Under controlled exposure settings, lungs from healthy, young adults 18–40 years of age were affected by WFS in a sex-dependent manner with males having upregulation of inflammatory genes and females having suppression of defense responses (41). Sex disparity was also observed in woodsmoke induced inflammation in lungs and brains in animal models (42, 43). However, we cannot exclude possibility that sex disparity observed in the LSC was due to more outdoor activities and higher exposure in males compared to females. Elevated susceptibility in people with less than college education may be due to their higher likelihood of WFS exposure and lower adoption of protective behaviors during wildfire smoke events. We also found that ever woodsmoke exposure for over a year potentiates the health impacts of WFS and this effect modification is independent of airway obstruction or baseline comorbidities. The biological mechanisms underlying the effect potentiation of WFS exposure warrant further investigation.

The findings of this study may be specific to populations in the Southwestern U.S., particularly those exposed to aged WFS originating from wildfires in the Gila, Apache, and Apache-Sitgreaves National Forests. The smoke traveled nearly 200 miles through the atmosphere before reaching the Albuquerque metropolitan area. The toxicity of WFS is influenced by the constituents present, which are determined by factors such as biomass fuel types, metals in the fuel, burning conditions, and secondary reactions during atmospheric transport. Future studies that analyze PM_2.5_ constituents in air samples collected during WFS events will provide further insight in associations observed in populations from smoke-prone regions.

In summary, our study suggests that episodic exposure to WFS PM_2.5_ and BC was associated with poorer respiratory-specific or general health-related quality of life, with notable differences in the temporal relationships between mental and physical health measures. Additionally, WFS PM_2.5_ exhibited higher toxicity than total ambient PM_2.5_, underscoring the importance of source-specific risk assessment for air pollution. Moreover, BC constituents may be more potent in inducing health effects compared to non-BC constituents in WFS PM_2.5_. Male participants, individuals with less than a college education, and those exposed to woodsmoke demonstrated heightened vulnerability to WFS. Understanding the factors driving these health disparities is essential for developing precision public health interventions to protect at-risk populations.

## Supplementary Material

Supplementary Files

This is a list of supplementary files associated with this preprint. Click to download.

• SupplementWildfireSmokeSGRQRR20250707.docx

## Figures and Tables

**Table 1:** Demographics of the Study Participants (n = 747)

Characteristics	All participants	7-day no WFS	7-day with WFS	*P* Value
	(N = 747)	(N = 469)	(N = 278)	
Age, mean (SD), yr	56.87 (9.08)	56.47 (9.03)	57.53 (9.13)	0.12
Sex, No. (%)				0.25
Male	364 (48.73)	221 (47.12)	143 (51.44)	
Female	383 (51.27)	248 (52.88)	135 (48.56)	
BMI, mean (SD), kg/m^2^	28.78 (6.35)	28.48 (5.88)	29.29 (7.06)	0.09
Ethnicity, No. (%)				0.08
Non-Hispanic	562 (75.23)	343 (73.13)	219 (78.78)	
Hispanic	185 (24.77)	126 (26.87)	59 (21.22)	
Education level, No. (%)				0.12
Less than college	268 (35.88)	178 (37.95)	90 (32.37)	
Some college or above	479 (64.12)	291 (62.05)	188 (67.63)	
Current smokers, No. (%)				0.20
No	300 (40.16)	180 (38.38)	120 (43.17)	
Yes	447 (59.84)	289 (61.62)	158 (56.83)	
Pack-years, mean (SD)	41.41 (20.96)	40.65 (20.44)	42.69 (21.80)	0.20
Woodsmoke, No. (%)				0.91
Never	506 (67.74)	317 (67.59)	189 (67.99)	
Ever	241 (32.26)	152 (32.41)	89 (32.01)	
CMH, No. (%)				0.13
No	518 (69.34)	316 (67.38)	202 (72.66)	
Yes	229 (30.66)	153 (32.62)	76 (27.34)	
Pulmonary disease, No. (%)				0.13
No	647 (86.61)	413 (88.06)	234 (84.17)	
Yes	100 (13.39)	56 (11.94)	44 (15.83)	

Abbreviation: BMI, Body Mass Index; CMH, chronic mucous hypersecretion; SD, standard deviation; WFS, wildfire smoke

**Table 2:** Descriptive Statistics of Wildfire Smoke Across Different Time Windows in the LSC Cohort

Exposure *	WFS PM_2.5_	WFS BC	Days affected by WFS	Area affected by WFS
7-day				
# of non-zero, %	278	278	278	278
Mean (SD)	0.37 (0.72)	0.02 (0.04)	2.75 (1.59)	17427 (22301)
Median (IQR)	0.03 (0.37)	0.0004 (0.01)	3.00 (3.00)	7436 (21201)
15-day				
# of non-zero, %	400	400	400	400
Mean (SD)	0.27 (0.58)	0.01 (0.03)	3.96 (3.12)	26726 (37558)
Median (IQR)	0.02 (0.22)	0.0005 (0.01)	3.00 (5.00)	9983 (30831)
30-day				
# of non-zero, %	540	540	540	540
Mean (SD)	0.20 (0.47)	0.01 (0.03)	5.69 (4.86)	37918 (54727)
Median (IQR)	0.03 (0.16)	0.006 (0.01)	4.00 (6.00)	14665 (46583)
60-day				
# of non-zero, %	613	613	613	613
Mean (SD)	0.17 (0.32)	0.01 (0.02)	9.80 (8.12)	67383 (89865)
Median (IQR)	0.02 (0.19)	0.0006 (0.01)	9.00 (11.00)	26155 (95921)

* PM_2.5_ and BC concentrations are measured in μg/m^3^. The number of days affected by smoke is reported in days, and the affected area is measured in km^2^. Means and medians are estimates among visits with any smoke days in defined time periods. WFS PM_2.5_ and WFS BC are calculated using the deviation methods with data from Wei et al as the input (6).

**Table 3:** Associations of Wildfire Smoke PM_2.5_ and BC with SGRQ Scores (N = 747) *

Exposure	Activity		Impacts		Symptom		Total	
	β (95%CI)	*P*	β (95%CI)	*P*	β (95%CI)	*P*	β (95%CI)	*P*
PM_2.5__7d (per 1 μg/m^3^)	1.03 (−0.28, 2.34)	0.122	0.52 (−0.27, 1.31)	0.195	1.29 (0.10, 2.48)	0.034	0.84 (−0.11, 1.79)	0.082
PM_2.5__15d (per 1 μg/m^3^)	1.23 (−0.14, 2.59)	0.079	0.78 (−0.04, 1.60)	0.064	1.42 (0.17, 2.67)	0.026	1.09 (0.10, 2.09)	0.031
PM_2.5__30d (per 1 μg/m^3^)	1.08 (−0.38, 2.54)	0.148	0.63 (−0.25, 1.51)	0.160	1.29 (−0.04, 2.63)	0.058	0.95 (−0.11, 2.02)	0.078
PM_2.5__60d (per 1 μg/m^3^)	0.59 (−1.02, 2.19)	0.473	0.36 (−0.60, 1.33)	0.463	0.87 (−0.60, 2.34)	0.245	0.59 (−0.57, 1.76)	0.319
WFS PM_2.5__7d (per 1 μg/m^3^)	5.51 (1.86, 9.16)	0.0032	3.27 (1.08, 5.47)	0.0035	6.15 (2.83, 9.48)	0.00031	4.68 (2.04, 7.33)	0.00053
WFS PM_2.5__15d (per 1 μg/m^3^)	5.34 (1.43, 9.24)	0.0075	3.16 (0.81, 5.50)	0.0086	4.88 (1.31, 8.45)	0.0075	4.38 (1.56, 7.21)	0.0024
WFS PM_2.5__30d (per 1 μg/m^3^)	4.63 (0.35, 8.91)	0.034	2.88 (0.30, 5.45)	0.029	3.40 (−0.52, 7.32)	0.090	3.72 (0.61, 6.82)	0.019
WFS PM_2.5__60d (per 1 μg/m^3^)	1.96 (−3.89, 7.81)	0.512	1.68 (−1.84, 5.19)	0.351	1.36 (−3.99, 6.72)	0.618	1.91 (−2.34, 6.15)	0.379
WFS PM_2.5__7d (per 0.05 μg/m^3^)	0.28 (0.09, 0.46)	0.0032	0.16 (0.05, 0.27)	0.0035	0.31 (0.14, 0.47)	0.00031	0.23 (0.10, 0.37)	0.00053
WFS PM_2.5__15d (per 0.05 μg/m^3^)	0.27 (0.07, 0.46)	0.0075	0.16 (0.04, 0.28)	0.0086	0.24 (0.07, 0.42)	0.0075	0.22 (0.08, 0.36)	0.0024
WFS PM_2.5__30d (per 0.05 μg/m^3^)	0.23 (0.02, 0.45)	0.034	0.14 (0.02, 0.27)	0.029	0.17 (−0.03, 0.37)	0.090	0.19 (0.03, 0.34)	0.019
WFS PM_2.5__60d (per 0.05 μg/m^3^)	0.10 (−0.19, 0.39)	0.512	0.08 (−0.09, 0.26)	0.351	0.07 (−0.20, 0.34)	0.618	0.10 (−0.12, 0.31)	0.379
WFS BC_7d (per 0.05 μg/m^3^)	5.31 (1.70, 8.92)	0.0041	3.19 (1.01, 5.36)	0.0041	6.11 (2.82, 9.40)	0.00029	4.55 (1.93, 7.16)	0.00070
WFS BC_15d (per 0.05 μg/m^3^)	5.38 (1.57, 9.18)	0.0057	3.09 (0.80, 5.38)	0.0084	4.99 (1.51, 8.47)	0.0051	4.37 (1.61, 7.13)	0.0020
WFS BC_30d (per 0.05 μg/m^3^)	4.87 (0.86, 8.87)	0.017	2.79 (0.38, 5.20)	0.024	3.60 (−0.07, 7.26)	0.055	3.75 (0.85, 6.66)	0.012
WFS BC_60d (per 0.05 μg/m^3^)	2.90 (−2.69, 8.50)	0.310	1.89 (−1.47, 5.26)	0.271	1.67 (−3.45, 6.80)	0.522	2.37 (−1.70, 6.43)	0.254
WFS days 7d (per 1 day)	0.57 (−0.49, 1.63)	0.290	0.41 (−0.22, 1.05)	0.205	1.21 (0.24, 2.17)	0.014	0.63 (−0.14, 1.40)	0.110
WFS days 15d (per 1 day)	0.22 (−0.35, 0.80)	0.453	0.26 (−0.09, 0.60)	0.142	0.45 (−0.08, 0.97)	0.094	0.30 (−0.12, 0.72)	0.157
WFS days 30d (perl day)	0.19 (−0.17, 0.55)	0.293	0.21 (−0.00, 0.43)	0.052	0.22 (−0.11, 0.55)	0.191	0.23 (−0.03, 0.49)	0.085
WFS days 60d (per) 1 day)	−0.08 (−0.30, 0.13)	0.437	0.01 (−0.12, 0.14)	0.916	−0.01 (−0.21, 0.19)	0.927	−0.02 (−0.18, 0.14)	0.812
WFS area 7d (per IQR)	0.36 (0.04, 0.67)	0.027	0.26 (0.07, 0.44)	0.0083	0.50 (0.22, 0.79)	0.00060	0.35 (0.12, 0.58)	0.0030
WFS area 15d (per IQR)	0.69 (−0.03, 1.41)	0.061	0.53 (0.10, 0.96)	0.017	0.87 (0.21, 1.53)	0.010	0.68 (0.16, 1.21)	0.011
WFS area 30d (per IQR)	0.62 (−0.46, 1.69)	0.260	0.65 (0.00, 1.30)	0.049	0.80 (−0.18, 1.78)	0.112	0.73 (−0.05, 1.51)	0.067
WFS area 60d (per IQR)	−0.74 (−2.68, 1.21)	0.458	0.24 (−0.94, 1.42)	0.689	0.22 (−1.58, 2.01)	0.811	−0.01 (−1.43, 1.41)	0.991

Abbreviations: β, regression coefficient; BC, black carbon; CI, confidence interval; PM_2.5_, particle with aerodynamic diameter ≤ 2.5 μm; PM_2.5__X d, average PM_2.5_ concentration during the X days prior to the interview; SGRQ, St. George's Respiratory questionnaire; WFS, Wildfire smoke.

* Models were adjusted for age, sex, ethnicity, BMI, education level, current smokers, pack-years, and prevalence of airway obstruction (defined as FEV1/FVC ratio ≤ 70%). WFS PM_2.5_ and WFS BC are calculated using the deviation methods with data from Wei et al as the input (6).

**Table 4:** Associations of Wildfire Smoke PM_2.5_ and BC with SF-36 Score (N = 747) *

Exposure	Mental Health Score		Physical Health Score	
	β (95%CI)	*P*	β (95%CI)	*P*
PM_2.5__7d (per 1 μg/m^3^)	−0.043 (−0.092, 0.006)	0.087	−0.063 (−0.111, −0.016)	0.0093
PM_2 5__15d (per 1 μg/m^3^)	−0.037 (−0.089, 0.014)	0.154	−0.068 (−0.118, −0.018)	0.0079
PM_2.5__30d (per 1 μg/m^3^)	−0.029 (−0.084, 0.026)	0.300	−0.063 (−0.117, −0.010)	0.020
PM_25__60d (per 1 μg/m^3^)	−0.005 (−0.065, 0.055)	0.866	−0.065 (−0.124, −0.007)	0.029
WFS PM_2.5__7d (per 1 μg/m^3^)	−0.172 (−0.309, −0.036)	0.014	−0.214 (−0.348, −0.081)	0.0016
WFS PM_2.5__15d (per 1 μg/m^3^)	−0.125 (−0.272, 0.022)	0.096	−0.192 (−0.335, −0.049)	0.0085
WFS PM_2.5__30d (per 1 μg/m^3^)	−0.075 (−0.236, 0.086)	0.361	−0.172 (−0.329, −0.016)	0.031
WFS PM_2.5__60d (per 1 μg/m^3^)	−0.035 (−0.254, 0.184)	0.754	−0.207 (−0.420, 0.006)	0.058
WFS PM_2.5__7d (per 0.05 μg/m^3^)	−0.009 (−0.015, −0.002)	0.014	−0.011 (−0.017, −0.004)	0.0017
WFS PM_2.5__15d (per 0.05 μg/m^3^)	−0.006 (−0.014, 0.001)	0.096	−0.010 (−0.017, −0.002)	0.0085
WFS PM_2.5__30d (per 0.05 μg/m^3^)	−0.004 (−0.012, 0.004)	0.361	−0.009 (−0.016, −0.001)	0.031
WFS PM_2.5__60d (per 0.05 μg/m^3^)	−0.002 (−0.013, 0.009)	0.754	−0.010 (−0.021, 0.000)	0.058
WFS BC_7d (per 0.05 μg/m^3^)	−0.172 (−0.307, −0.036)	0.013	−0.221 (−0.353, −0.089)	0.0011
WFS BC_15d (per 0.05 μg/m^3^)	−0.135 (−0.278, 0.008)	0.065	−0.201 (−0.341, −0.062)	0.0048
WFS BC_30d (per 0.05 μg/m^3^)	−0.100 (−0.250, 0.050)	0.193	−0.184 (−0.330, −0.038)	0.014
WFS BC_60d (per 0.05 μg/m^3^)	−0.075 (−0.284, 0.135)	0.486	−0.225 (−0.429, −0.021)	0.031
WFS days_7d (per 1 day)	−0.042 (−0.081, −0.002)	0.040	−0.043 (−0.081, −0.004)	0.030
WFS days_15d (per 1 day)	−0.015 (−0.036, 0.007)	0.179	−0.021 (−0.042, −0.000)	0.048
WFS days_30d (per 1 day)	−0.011 (−0.024, 0.002)	0.110	−0.017 (−0.030, −0.004)	0.012
WFS days_60d (per 1 day)	−0.001 (−0.009, 0.007)	0.730	−0.006 (−0.014, 0.001)	0.101
WFS area_7d (per IQR)	−0.015 (−0.027, −0.004)	0.011	−0.017 (−0.028, −0.005)	0.0041
WFS area_15d (per IQR)	−0.023 (−0.050, 0.004)	0.096	−0.032 (−0.059, −0.006)	0.017
WFS area_30d (per IQR)	−0.013 (−0.053, 0.027)	0.523	−0.038 (−0.077, 0.001)	0.058
WFS area_60d (per IQR)	0.008 (−0.065, 0.080)	0.837	−0.043 (−0.113, 0.027)	0.228

Abbreviations: β, regression coefficient; BC, BC; CI, confidence interval; PM_2.5_, particle with aerodynamic diameters ≤ 2.5 μm; PM_2.5__X d, average PM_2.5_ concentration during the X days prior to the interview; SF-36, the short form 36 health survey questionnaire; WFS, Wildfire smoke.

* Models were adjusted for age, sex, ethnicity, BMI, education level, current smokers, pack-years, and prevalence of comorbidity. WFS PM_2.5_ and WFS BC are calculated using the deviation methods with data from Wei et al as the input (6).

## Data Availability

De-identified data are available upon execution of a Data Use Agreement with Lovelace Biomedical Research Institute, the data owner.

## Abbreviations

BC: black carbon
COPD: chronic obstructive pulmonary disease
CMH: chronic mucous hypersecretion
HRQoL: health-related quality of life
LSC: the Lovelace Smokers Cohort
PM_2.5_: fine particulate matter
SF-36: the 36-Item Short Form Survey
SGRQ: The St. George’s Respiratory Questionnaire
WFS: wildfire smoke
